# *Trichoderma paratroviride* Strain 8942: Mechanisms of *Phytophthora infestans* Inhibition and Tomato Growth Promotion

**DOI:** 10.3390/jof12020096

**Published:** 2026-01-30

**Authors:** Hao Hu, Ting Huang, Heng-Xu Wang, Zhao-Qing Zeng, Wen-Ying Zhuang

**Affiliations:** 1 State Key Laboratory of Microbial Diversity and Innovative Utilization, Institute of Microbiology, Chinese Academy of Sciences, Beijing 100101, China; hwsw221@163.com (H.H.); zengzq@im.ac.cn (Z.-Q.Z.); 2 University of Chinese Academy of Sciences, Beijing 100049, China; 3 College of Grassland Science and Technology, China Agriculture University, Beijing 100193, China; h_ting17@163.com; 4 Institute of Soil Science, Chinese Academy of Sciences, Nanjing 211135, China; wanghx1127@163.com

**Keywords:** biocontrol, growth promotion, tomato late blight, transcriptome, *Trichoderma*

## Abstract

Tomato late blight caused by *Phytophthora infestans* is a devastating disease, and current control of the disease relies heavily on chemical fungicides. Certain *Trichoderma* strains used as biocontrol fungi have shown superb efficacy against *P. infestans* and some other oomycete phytopathogens. In this study, *T. paratroviride* strain 8942 appeared to be effective in control of tomato late blight disease, reducing the necrosis degree of plant tissues, promoting callose deposition in tomato leaves, and increasing defense enzyme activities. RT-qPCR analysis showed that strain 8942 inhibited metabolism of salicylic acid and promoted metabolism of jasmonic acid at the early stage of colonization. In addition, root colonization of the strain significantly promoted tomato growth. Observations of rhizosphere soil properties showed that 8942 significantly increased the activities of urease, catalase, and protease, and its cell-free filtrates at low concentrations induced the accumulation of auxin in root tips. Transcriptomic data suggested the existence of a balance between biotrophic adaptation and biocontrol readiness during 8942’s interaction with tomato roots. *Trichoderma paratroviride* strain 8942 is promising and has potential for biological control of tomato late blight and plant growth promotion, as determined by integrated investigations of hormonal regulation, rhizosphere modulation, transcriptional reprogramming, etc.

## 1. Introduction

Late blight, caused by the oomycete pathogen *Phytophthora infestans*, poses a severe threat to global tomato production due to its rapid spread, high virulence, and limited sustainable management strategies, and challenges like pathogen resistance and environmental pressure from chemical fungicides have to be faced [1,2]. Recognized as the most destructive oomycete pathogen [2], *P. infestans* has driven an urgent need for eco-friendly biocontrol agents. *Trichoderma*, a genus of filamentous fungi renowned for their antagonistic activity against fungal phytopathogens, has shown promise in this regard [3,4]. However, its efficacy against oomycetes, particularly *P. infestans*, remains poorly understood, and physiological, molecular, and transcriptomic studies on *Trichoderma*–oomycete interactions are lacking. Only some strains of a limited number of species like *T. atroviride*, *T. koningiopsis*, *T. hamatum*, and *T. asperellum* have been reported to demonstrate certain biocontrol effects [4,5,6].

*Trichoderma* strains deploy multifaceted mechanisms to suppress phytopathogens, including mycoparasitism, nutrient competition, and antibiosis [7,8]. During mycoparasitism, *Trichoderma* hyphae recognize, coil around, and penetrate phytopathogen structures; secrete cell wall-degrading enzymes (CWDEs) such as chitinases, cellulases, xylanases, and proteases to dismantle the pathogen’s cell wall; and facilitate nutrient acquisition [9,10]. Unlike fungi, oomycete cell walls are primarily composed of cellulose, which suggests that CWDEs secreted during *Trichoderma*–oomycete interactions may have different patterns [11]. Concurrently, *Trichoderma* strains produce a diverse array of antimicrobial secondary metabolites (SMs), including gliotoxins, lipopeptides, steroids, polyketides, terpenes, and phenolic compounds, which inhibit pathogen growth [9,12]. These compounds, coupled with rapid hyphal growth and sporulation, enable *Trichoderma* to dominate ecological niches and outcompete nutrition resources [13].

Through long-term co-evolution, *Trichoderma* strains developed sophisticated mechanisms to asymptomatically colonize plant tissues, establish persistent beneficial interactions, and induce plant physiological and defense responses [14,15]. Following colonization, *Trichoderma* strains trigger physical or biochemical reactions in plants and confine their growth superficially or intercellularly within cortical tissues [16]. These interactions have been shown in model or other pathosystems to induce plant defense responses, including deposition of cell wall-associated compounds (e.g., lignin and callose), production of reactive oxygen species (ROS) and reactive nitrogen species, and synthesis of antimicrobial SMs [17,18,19,20]. The secondary metabolites identified in recent studies, such as hemiterpenes and polyketides, in *T. arundinaceum*, *T. atroviride*, and *T. viride*, specifically induce plant resistance [21,22]. Additionally, analysis of early interactions between *T. hamatum* T382 and *Arabidopsis thaliana* revealed the *Trichoderma*-induced changes in the activities of peroxidases (POD), glutathione reductase, glutathione S-transferases, and other detoxification enzymes in leaves [23]. Beneficial microbe–plant interactions are closely linked to induced systemic resistance (ISR), which involves the signaling pathways related to hormones such as salicylic acid (SA), jasmonic acid (JA), and ethylene [24]. The specific roles of *Trichoderma* in tomato–*P. infestans* interactions remain to be explored.

Microorganisms closely associated with plant roots directly influence plant growth and development by modifying rhizosphere soil physicochemical properties and stimulating root system growth [25,26]. *Trichoderma* strains enhance nutrient solubility in the soil, facilitate plant nutrient uptake (particularly nitrogen) in the rhizosphere, improve phosphate acquisition by solubilizing diverse phosphate sources, and thereby promote plant growth. They also induce soil acidification, alter soil enzyme activities (e.g., urease and phosphatase), and modify root architecture [27]. Recent studies suggest that auxin-mediated responses may contribute to *Trichoderma*-induced root growth promotion. Plant mutants defective in the related pathways exhibit reduced *Trichoderma*-associated effects [28,29].

Transcriptomic studies have elucidated key genetic and metabolic adaptations of *Trichoderma*’s plant growth promotion and biocontrol efficacy. Interactions between *Trichoderma* strains and plants may modulate transcriptional responses: *T. asperellum* AC30536 upregulated heat shock proteins and glycosyl hydrolases when colonizing tomato roots [30], and *T. virens* differentially expressed transporters and small secreted proteins (SSPs) in maize rhizospheres [31]. As far as mycoparasitism is concerned, *T. atroviride* SZMC 24276TA upregulated SSPs with CAP and CFEM domains, which highlights their role in immune modulation [32]. The above studies collectively underscore *Trichoderma*’s transcriptional plasticity in plant pathogen suppression, symbiotic interaction, and metabolic resource allocation.

In this study, *T. paratroviride* strain 8942 (data unpublished, abbreviated as 8942 in the following text) was found to not only suppress tomato late blight but also enhance plant growth. Our work intends to provide critical insights into physiological/molecular mechanisms of *Trichoderma*-mediated biocontrol and to determine the potential of 8942 in tomato late blight management.

## 2. Materials and Methods

### 2.1. Trichoderma Strain, Phytopathogen, and Plant Materials

*Trichoderma paratroviride* 8942 was preserved in the State Key Laboratory of Microbial Diversity and Innovative Utilization, Institute of Microbiology, Chinese Academy of Sciences, and the China General Microbiological Culture Collection Center (CGMCC No. 3.29720). The strain was maintained on potato dextrose agar (PDA; 200 g potato, 20 g glucose, 20 g agar per L) at 25 °C. The oomycete pathogen *Phytophthora infestans* strain PP34 (T30-4, referred to as PP34 in the following text) was kindly provided by Prof. Suo-Meng Dong. It was cultured on oatmeal-V8 agar (OA-V8; 30 g oatmeal, 20 g glucose, 20 g agar, 10% V8 juice, 1 g CaCO_3_/L) at 18 °C. Tomato (*Solanum lycopersicum* cv. ZhongZa 9) seeds were obtained from China Vegetable Seed Technology Co., Ltd. (Beijing, China), and *Arabidopsis thaliana* DR5::GFP lines were provided by Prof. Tong-Da Xu.

### 2.2. Biocontrol Efficacy of Trichoderma paratroviride Strain 8942

The antagonistic activity of *T. paratroviride* 8942 against *P. infestans* PP34 was tested using a dual-culture assay [6]. Mycelial plugs (5 mm diameter) from actively growing colonies of 8942 and PP34 were placed 6 cm apart on individual OA-V8 plates (9 cm diameter). Strain PP34 was inoculated 4 days ahead of 8942 due to its slow growth. Plates inoculated with PP34 alone served as a control. All treatments were incubated at 18 °C in darkness, with three replicates. Hyphal interactions of the two strains were examined 24–48 h after colonies contacted each other by cutting 5 × 5 mm mycelial blocks and were observed under an optical microscope (Zeiss Imager A2, Jena, Germany).

Cell-free filtrate (CFF) and volatile organic compounds (VOCs) produced by 8942 were used to test its abilities in vitro against PP34 [6,33]. A spore suspension (1 × 10^7^ spores/mL) of 8942 from a 7-day PDA culture was inoculated into potato dextrose broth (PDB, 1% *v*/*v*) and incubated at 28 °C for 7 days at an agitation speed of 180 rpm. CFF was obtained by filtering supernatants through a 0.22 μm membrane. Strain PP34 mycelial plugs were inoculated onto OA-V8 plates containing 10% CFF (*v*/*v*) with sterile PDB as a control. To evaluate the anti-*Phytophthora* effect of the VOCs produced by 8942, the PP34-containing plates were inverted and placed on the top of the plates with 8942, then sealed with parafilm to allow only volatile compounds to affect the culture of PP34 and incubated at 18 °C for 7 days. The plates lacking *Trichoderma* served as a control. The growth radius of PP34 was measured.

Tomato seedlings were cultivated in pots containing a mixture of peat and vermiculite (2:1, *v*/*v*) and maintained under a cycle of 16/8 h (light/dark) at 26 °C. Four-leaf-stage healthy plants were selected and sprayed with 10 mL of a spore suspension (1 × 10^7^ spores/mL) of 8942, and seedlings treated with sterile water served as a control. Each treatment group containing six plants was incubated in darkness for 24 h. Then, a zoospore suspension of PP34 (5 × 10^4^ zoospores/mL) was sprayed and maintained at 20 °C with 75–80% relative humidity under a cycle of 12/12 h light/dark. Disease severity was scored 7 days post-inoculation (dpi) using a 0–5 scale measurement (Figure S1, 0: no symptoms; 5: complete necrosis) [4,34]. Relative disease indexes (RDis) were calculated separately as follows: RDi = ∑ (Di × Li)/(L × N) × 100 [Di = disease severity grade, Li = number of leaflets at grade Di, L = total leaflets, N = number at the highest grade of disease severity (grade 5)].

### 2.3. Induced Defense Responses by T. paratroviride 8942 in Tomato Against Late Blight

Tomato plants treated with 8942 (see Section 2.2) were analyzed for physiological and molecular defense responses to PP34 infection. Leaf samples from similar layer across different plants were collected destructively at pre-treatment and the 1st, 3rd, 5th, and 7th days post-inoculation with PP34. Individual treatments were repeated three times. For measurements of callose deposition, stomatal aperture, and tissue staining, six leaves were selected in each treatment. For defense enzyme activity and gene expression analysis, ca. 2 g of leaf tissues per treatment was sampled. Evaluation parameters consisted of physical defenses, photosynthesis, antioxidant enzyme activities, and expression of defense-related genes (Table S1).

### 2.4. Plant Growth Promotion Ability of T. paratroviride 8942

Seeds of tomato and *A. thaliana* DR5::GFP were sown by conventional methods (see Section 2.2). At the 2–3-true-leaf stage, roots were inoculated with 10 mL spore suspension of 8942 (1 × 10^7^ spores/mL), and those treated with sterile water served as a control. Each treatment contained 6 plants. Plant height, fresh weight, and dry weight were measured 3 weeks after inoculation. Auxin accumulation in *A. thaliana* DR5::GFP root tips was visualized by GFP fluorescence (see Section 2.5).

Cell-free filtrate of 8942 (see Section 2.2) was diluted to 1%, 5%, 10%, and 20% (*v*/*v*) and incorporated into MS medium (Beijing Solarbio Science & Technology Co., Ltd., Beijing, China). Absence of CFF served as a control. *Arabidopsis thaliana* DR5::GFP seeds were surface-sterilized (treated with 3% NaClO for 30 s, 75% ethanol for 1 min, then washed with sterile water three times) and sown on MS media containing different concentrations of CFF and the control substrate. Each treatment contained six seedlings, and was repeated three times. The samples were cultured at 25 °C under a cycle of 12/12 h day/night, and the root length, lateral root number (calculated via Photoshop 2021), and auxin distribution (quantified using Fiji ImageJ 1.54f [http://imagej.nih.gov/ij/ (accessed on 11 May 2025)] were recorded after 7 days.

### 2.5. Examination of A. thaliana Roots in Contact with T. paratroviride 8942

Root samples were rinsed with sterile water, and residual water on root surfaces was blotted with absorbent paper. The samples were stained with 10 mg/mL propidium iodide (PI; prepared with 1 × PBS, 0.02% Tween 20) for 1 min, fixed on a microscope slide in 50% (*v*/*v*) glycerol, and examined using a laser confocal microscope (CLSM, Leica SP8, Leica Microsystems, Wetzlar, Germany) with excitation/emission wavelengths of 488/500–550 nm (GFP) and 561/570–655 nm (PI). Twelve root tips were randomly selected from individual treatments. Fluorescence signal intensities in all images were quantified using Fiji ImageJ 1.54f.

### 2.6. Rhizosphere Soil Physicochemical Properties Influenced by T. paratroviride 8942

Rhizosphere soil samples of the different treatments were collected from tomato plants after 21 days by gently shaking the roots to remove loosely adhered soil and then scraping tightly bound soil with a sterile spatula. The samples were taken from 3 cm and 6 cm below the rhizosphere soil surface of the pots, then homogenized and air-dried [35]. Relevant physicochemical parameters of the rhizosphere soil samples were determined, which included total nitrogen, total phosphorus, neutral phosphatase, urease, protease, and catalase [36,37]. The plant growth promotion mechanism of 8942 was evaluated.

### 2.7. Transcriptomic Analysis of Interactions Between T. paratroviride 8942 and Tomato

Following the methods mentioned by Romero-Contreras et al. [38] and Zhang et al. [39], tomato seeds were surface-sterilized, with 6 seeds put on one side of each 9 cm diameter MS plate, and co-cultured with 8942 on the other side of the plate after germination of the seeds. The samples treated with 8942 were collected 24 h after hyphae contacting tomato roots (Figure S2). Root samples that had not yet contacted 8942 served as a control. All treatments were of three replications. They were all flash-frozen in liquid nitrogen and stored at −80 °C. RNA sequencing and differential gene expression analysis were performed by Beijing BerryGenomics Co., Ltd. (Beijing, China).

### 2.8. Real-Time qPCR Analysis

Total RNA was extracted using the RNAprep Pure Plant Kit (TIANGEN Biotech. Co., Ltd., Beijing, China). cDNA was synthesized from RNA using the RNA HiScript III RT SuperMix kit (Vazyme Biotech Co., Ltd., Nanjing, China) and amplified via ChamQ Universal SYBR qPCR Master Mix (Vazyme). The RT-qPCR reaction system (20 μL) consisted of 1 μL cDNA, 10 μL 2 × ChamQ Universal SYBR qPCR Master Mix, 1 μL upstream/downstream primer (10 μM), and 7 μL ddH_2_O. The reaction procedure was 95 °C for 2 min, followed by 95 °C for 20 s, 56 °C for 30 s, and 72 °C for 40 s (40 cycles), which was repeated three times. Relative gene expression was calculated using the 2^−ΔΔCq^ method [39]. Primer sequences are listed in Table S2.

### 2.9. Statistical Analysis

Assumptions of normality and homogeneity of variance were first tested. Normality was assessed with the Shapiro–Wilk test, and homogeneity of variance was evaluated by Levene’s test. Gene expression of the transcriptome was quantified using FPKM values. FeatureCount was used for gene-level quantification of each sample, and differential expression analysis was performed via edgeR 3.3.3. The specific analysis parameters were as follows: |log2 (fold change)| > 1.0 and q-value < 0.05. Data are presented as means ± SDs. Differences between treatments were assessed using one-way ANOVA followed by Duncan’s multiple-range test (*p* ≤ 0.05) in SPSS Statistics 20 (IBM, Armonk, NY, USA).

## 3. Results

### 3.1. Biocontrol Effects of T. paratroviride 8942 on P. infestans PP34

**Figure 1:**
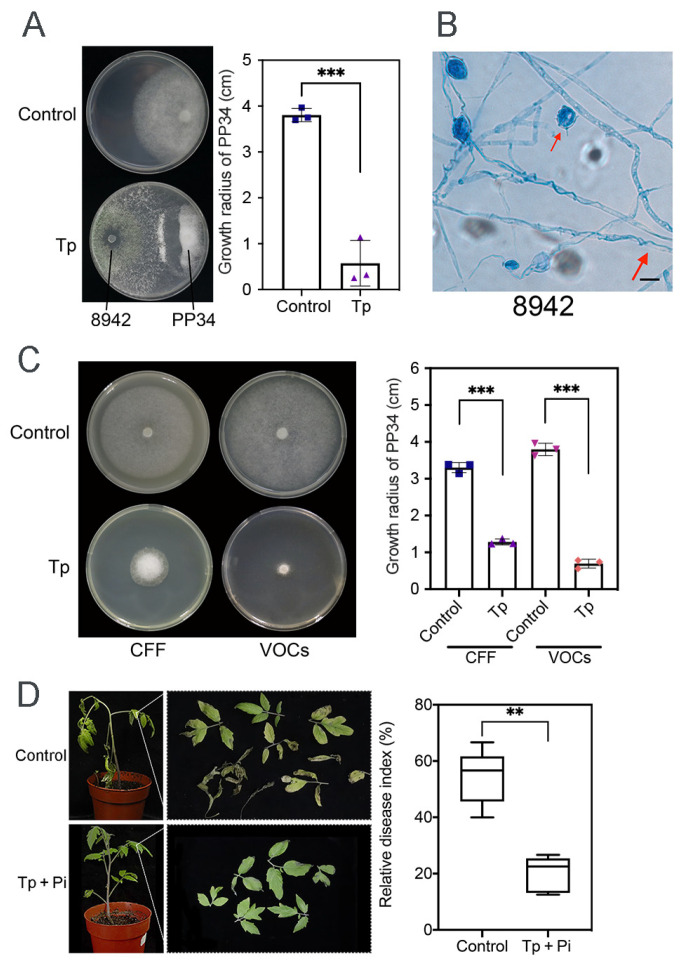
Antagonistic activity of *T. paratroviride* 8942 against *P. infestans* PP34. (**A**) Interactions between *T. paratroviride* 8942 and *P. infestans* PP34 in dual culture (Tp: *Trichoderma* treatment; Control: PP34 only). (**B**) Hyphal coilings (red arrow) of 8942 surrounding hyphae and zoosporangia of PP34 under light microscopy. Bar = 20 μm. (**C**) Growth-inhibitory effects of 8942’s cell-free filtrate (CFF) and volatile organic compounds (VOCs) on PP34. (**D**) Biocontrol efficacy of 8942 on PP34 (Tp + Pi: inoculated with *Trichoderma* and PP34; Control: PP34 only). Data are shown as means ± SDs. Significance: ** 0.001 < *p* ≤ 0.01, *** *p* ≤ 0.001.

Compared with the control, dual-culture assays of 8942 against PP34 revealed a strong inhibition ability (85%), in which hyphal coilings of 8942 surrounding hyphae and zoosporangia of PP34 were commonly observed (Figure 1A,B). CFF inhibitory activity and the effects of VOCs of 8942 on PP34 were also evaluated. The results showed that CFF and VOCs of 8942 significantly inhibited the growth of PP34 (>70%), which highlights the functions of both soluble and volatile antimicrobial metabolites in *Trichoderma*-mediated pathogen suppression, which were certainly different in the control treatments (Figure 1C). Pot experiments (n = 6) with 8942 were further evaluated to validate the strain’s efficacy in terms of control of tomato late blight. The results revealed that 8942 reduced the relative disease index by 20.42% (Figure 1D).

### 3.2. Tomato Defense Responses Activated by T. paratroviride 8942

**Figure 2:**
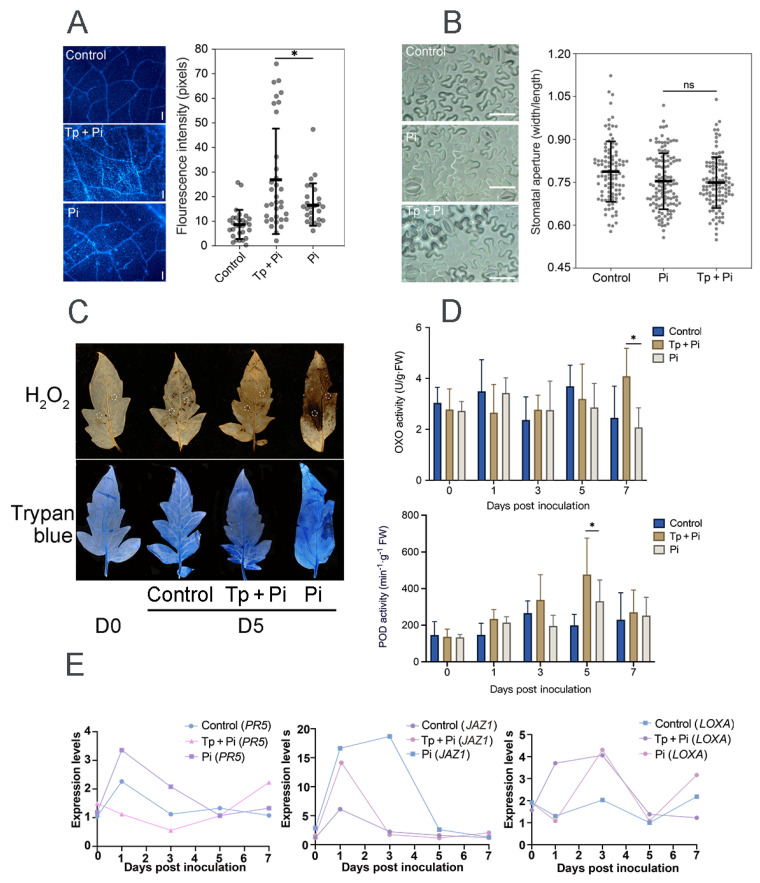
Defense responses in tomato induced by *T. paratroviride* 8942 against *P. infestans* PP34. (**A**) Callose deposition in tomato leaves at the 5th day post-inoculation (dpi) detected by aniline blue staining (Tp + Pi: 8942 and PP34; Pi: PP34 only; Control: sterile water); bar = 50 µm. (**B**) Stomatal aperture size of leaves at the 5th dpi; bar = 20 µm. (**C**) Histochemical detection of H_2_O_2_ (DAB staining) and visualization of cell death (trypan blue staining) of leaves at the 5th dpi; circles on leaves indicating areas callose deposited. (**D**) Activities of oxalate oxidase (OXO) and peroxidase (POD) in leaves. (**E**) Relative expression levels of defense-related genes in jasmonic acid (JA; *LOXA* and *JAZ1*) and salicylic acid (SA; *PR5*) signaling pathways. Leaf samples were collected with pre-treatment of PP34 at the 0, 1st, 3rd, 5th, and 7th dpi. Significance: * 0.01 < *p* ≤ 0.05, ns: not significant.

Physical barriers, such as callose (a β-1,3-glucan polymer), provide a primary line of plant defense against pathogens. Using aniline blue staining coupled with fluorescence microscopy, callose deposition patterns in tomato leaves were assessed. For each sample, 10–12 microscopic fields were examined, and the statistical analysis indicated significant differences (Figure 2A). The results demonstrated that 8942 significantly stimulated callose accumulation in all leaf sections compared with the control. The fluorescence signal intensity of the 8942-treated leaves was 27.2 pixels, while that of the PP34 treatment was 17.1 pixels (Figure 2A). Stomata serve as essential paths for CO_2_ and H_2_O exchange and are entry points for pathogen invasion, which becomes a critical factor influencing plant–pathogen interaction. However, our data showed that 8942 did not obviously alter the stomatal aperture size of tomato leaves (Figure 2B), which suggests that *Trichoderma* 8942-mediated tomato defense mechanisms might not be involved in stomatal regulation.

Trypan blue staining was employed to quantify necrosis levels of tomato leaves infected by strain PP34. Our results found that co-inoculation of 8942 and PP34 significantly mitigated leaf necrosis compared with the PP34-alone treatment (Figure 2C) and effectively alleviated oxidative stress in plant tissues by reducing ROS accumulation, specifically hydrogen peroxide (H_2_O_2_) levels (Figure 2C), which suggests that 8942 enhanced the disease resistance of tomato, as well as the positive reaction of the plant to environmental stresses. To further characterize the defense responses, the activities of oxalate oxidase (OXO) and POD were measured. Along with the inoculation, OXO activity decreased progressively in the PP34-treated plants. In contrast, the *Trichoderma* treatment increased plant OXO activity, the OXO activity being significantly higher than that of the control at the 7th day post-inoculation (Figure 2D). POD activity showed an initial surge, followed by downregulation across all treatments. However, the 8942-treated plants displayed a substantially higher POD peak at 5 dpi, which is 1.4-fold that of the control; in other words, the ROS detoxification capacity of tomato was enhanced (Figure 2D).

Plant resistance mechanisms include systemic acquired and induced systemic resistance, which are mediated by the SA and JA signaling pathways, respectively. Based on the gene expression analysis at different time points, our results showed that the 8942 treatment significantly upregulated gene expression associated with the ISR pathway *LOXA* but downregulated *JAZ1* at the early stage of colonization, and gene expression in relation to the SAR pathway *PR5* was downregulated (Figure 2E).

### 3.3. Enhancement of Plant Growth by T. paratroviride 8942

**Figure 3:**
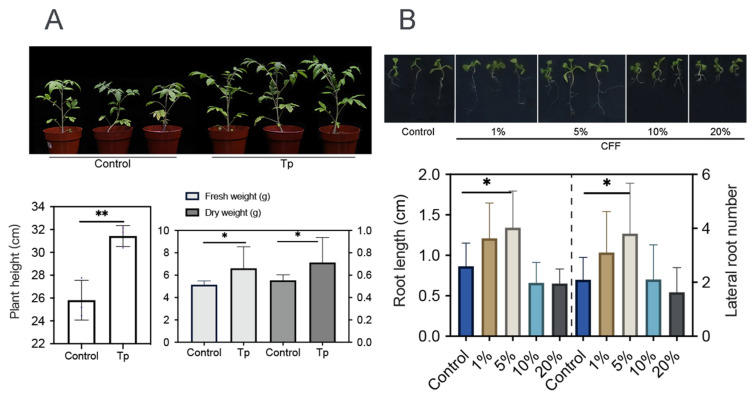
Growth promotion effects of *T. paratroviride* 8942 on tomato and *Arabidopsis thaliana*. (**A**) Biomass parameters (plant height, fresh weight, and dry weight) of tomato 3 weeks after inoculation with *T. paratroviride* 8942 (Tp: treated with spore suspension of 8942; Control: treated with sterile water). (**B**) Influences of 8942 cell-free filtrate concentrations (1%, 5%, 10%, and 20%) on root architecture in *Arabidopsis thaliana.* Significance: * 0.01 < *p* ≤ 0.05, ** 0.001 < *p* ≤ 0.01.

Beneficial microbes, including some *Trichoderma* strains, are able to promote plant growth [10,40]. In this work, 8942’s ability was tested through root colonization. It appeared that the strain enhanced tomato growth with the increase in height, fresh weight, and dry weight (Figure 3A). It was also discovered that low concentrations (1% and 5%) of CFF promoted root elongation and lateral root development of *A. thaliana* (Figure 3B).

### 3.4. Rhizosphere Modulation and Auxin Accumulation Influenced by T. paratroviride 8942

**Figure 4:**
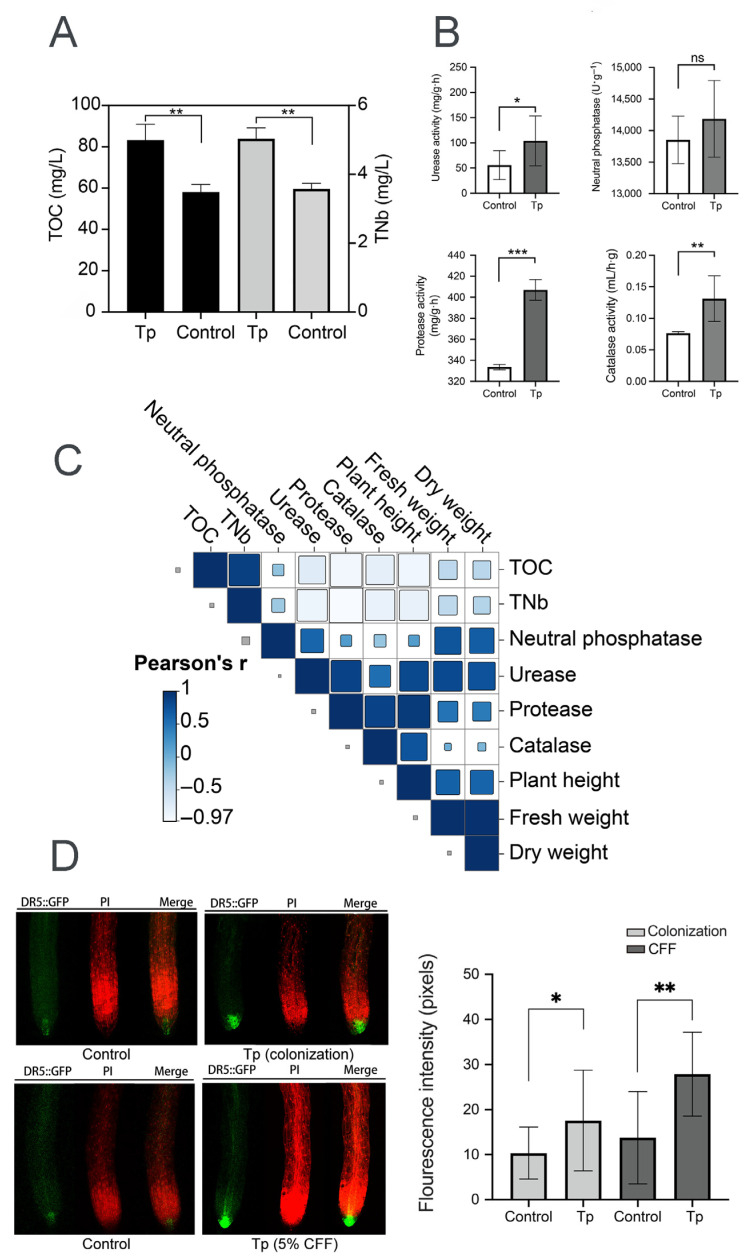
Rhizosphere modulation and auxin accumulation influenced by *T. paratroviride* 8942. (**A**) Nutrient content (TOC: total organic carbon; TNb: total nitrogen) and (**B**) activities of enzymes (urease, neutral phosphatase, protease, and catalase) in rhizosphere soil after *T. paratroviride* 8942 colonization (Tp: treated with 8942 spore suspension; Control: treated sterile water). (**C**) Correlation analysis of plant growth parameters, soil nutrients, and soil enzyme activities. r: Pearson’s correlation coefficient of color scale. (**D**) Auxin accumulation visualized in root tips of *Arabidopsis thaliana* DR5::GFP (green fluorescence: GFP; red fluorescence: propidium iodide). Significance: * 0.01 < *p* ≤ 0.05, ** 0.001 < *p* ≤ 0.01, *** *p* ≤ 0.001, ns: not significant.

To explore mechanisms underlying the effects of strain 8942 on plant growth, soil physicochemical properties and auxin accumulation in plant roots were analyzed. Strain 8942 increased the concentrations of total organic carbon and total nitrogen (Figure 4A). The activities of 8942’s colonization elevated urease by 1.8-fold, protease by 1.2-fold, and catalase by 1.7-fold in the rhizosphere soil (Figure 4B). Notably, urease and protease were correlated positively with plant biomass (plant height, fresh weight, and dry weight). For example, the Pearson correlation coefficients for urease with plant height, fresh weight, and dry weight were 0.81, 0.82, and 0.74, respectively, and those for protease were 0.92, 0.48, and 0.43 (Figure 4C). Furthermore, auxin accumulation in tomato root tips was enhanced by colonization of the strain and a low concentration of CFF. A 2.03-fold increase in auxin signal accumulation was observed in *A. thaliana* DR5::GFP root tips of 8942’s CFF (Figure 4D).

### 3.5. Transcriptome Analysis of Interactions Between T. paratroviride 8942 and Tomato

**Figure 5:**
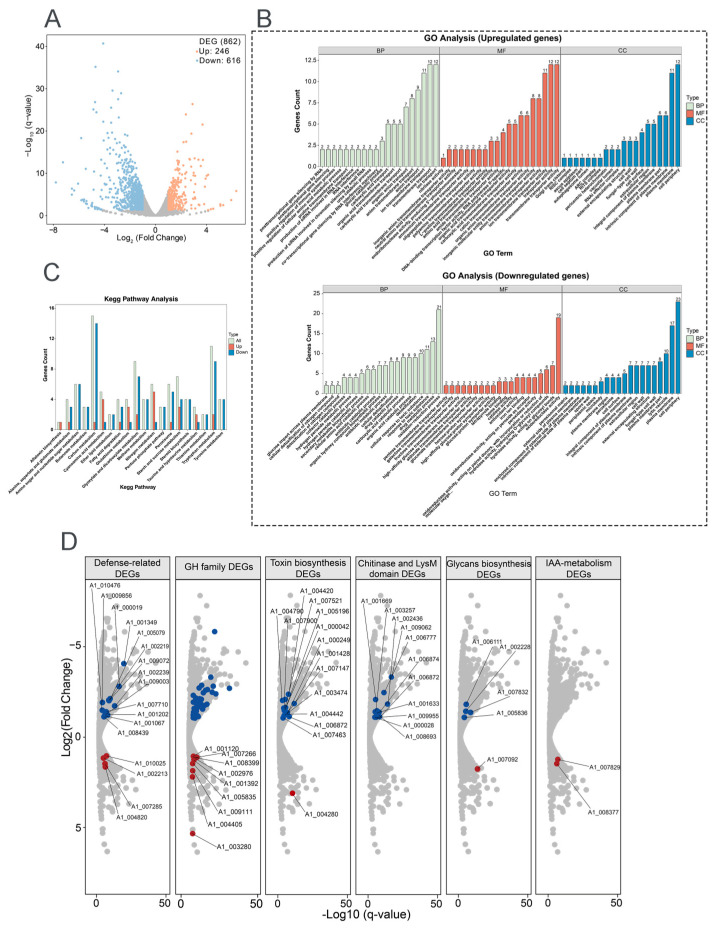
Transcriptomic profiling of *T. paratroviride* 8942 interaction with tomato. (**A**) Volcano plot of differentially expressed genes (DEGs); false discovery rate (FDR) ≤ 0.05, fold change (FC) ≥ 2 or ≤0.5 as criteria for identification of DEGs. (**B**) Gene Ontology (GO) enrichment and (**C**) Kyoto Encyclopedia of Genes and Genomes (KEGG) for pathway analysis of DEGs. BP: biological process; CC: cellular component; MF: molecular function. (**D**) Expression patterns for key DEGs involved in fungal cell wall degradation, secondary metabolism, defense, and extracellular structure remodeling (red dots: upregulated genes; blue dots: downregulated genes); GH: glycoside hydrolase (blue dots: forty downregulated genes); IAA: Indole-3-acetic acid.

Transcriptome analysis of 8942 interacting with tomato roots revealed significant differential gene expression compared with the control. A total of 862 genes were differentially expressed (DEGs; |log2FC| ≥ 1, FDR < 0.05); among them 246 were upregulated and 616 genes were downregulated at the early stage of root colonization, which gives the hint that dynamic reprogramming of *Trichoderma* metabolism happened during symbiosis (Figure 5A).

Gene Ontology (GO) enrichment analysis demonstrated that differentially expressed genes were predominantly related to three categories, i.e., biological process (BP), cellular component (CC), and molecular function (MF). Upregulated genes were enriched in transmembrane transport, ion transport, anion transport (BP category), plasma membrane, cell periphery (CC category), transporter activity, anion transmembrane transporter activity, ion transmembrane transporter activity, and organic anion transporter (MF category) (Figure 5B). Downregulated genes were enriched in the oxidation–reduction process, response to toxic substances, the drug metabolic process (BP category), cell peripheries, plasma membranes, fungal cell walls, lytic vacuoles (CC category), and oxidoreductase activity (MF category) (Figure 5B). KEGG pathway analysis further emphasized the activations of the related metabolism pathways, carbon, tryptophan, glyoxylate and dicarboxylate, starch, and sucrose (Figure 5C).

To determine key functional properties involved in ISR and growth promotion, the gene expressions of fungal cell wall-degrading enzymes (FCWDEs), SMs, defense, and extracellular structure remodeling were analyzed after 8942 contacting tomato. For FCWDEs, 40 glycoside hydrolase genes (GH family) were downregulated. Repression of specific FCWDEs indicated that these enzymes may be linked to avoidance of plant defenses; however, nine other genes in the GH family were enhanced during colonization (Figure 5D). SMs played widely acknowledged roles in plant growth and disease resistance. In relation to toxin biosynthesis, 14 genes were downregulated and 1 was upregulated. Meanwhile, two genes related to indole-3-acetic acid (IAA) metabolism were upregulated (Figure 5D). Among the defense-related genes, six glutathione S-transferase genes were significantly repressed during recognition, and three were upregulated. Five catalase genes and six peroxidase genes were downregulated, and one peroxidase gene was upregulated (Figure 5D). For extracellular structure remodeling, chitin and glycans serve as microbial- or pathogen-associated molecular patterns recognized by plants. Ten chitinase genes were downregulated, and one LysM-domain gene was downregulated. Four glycan biosynthesis genes were downregulated, and one glycan degradation gene was upregulated (Figure 5D).

## 4. Discussion

Currently, late blight disease control relies heavily on chemical fungicides, the application of which faces the challenges of phytopathogen resistance and environmental pollution. The potential of *Trichoderma* strains as opportunistic plant symbionts for control of diseases caused by oomycetes, particularly *P. infestans*, remains unclear. The related research on biocontrol potentials or mechanisms against oomycete phytopathogens appears to be limited. Previous studies concentrated mostly on strains of a few *Trichoderma* species, like *T. atroviride*, *T. viride*, *T. koningiopsis*, and *T. asperellum* [5,6]. Our work demonstrated that *T. paratroviride* strain 8942 displayed strong biocontrol activity against *P. infestans* PP34 and plant growth promotion ability, as supported by multifaceted physiological and molecular evidence. The discovery of novel *Trichoderma* resources to deal with tomato late blight management should be given the attention it deserves.

Multifaceted biocontrol mechanisms of *Trichoderma* strains involve direct phytopathogen inhibition and induced plant disease resistance [41]. In this study, a dual-culture assay showed that strain 8942 inhibited more than 80% of *P. infestans* mycelial growth (Figure 1A). This level of suppression is comparable to the inhibition range (75–91%) reported for high-performing *Trichoderma* strains against *Phytophthora* recorded in previous studies [5,6]. Strain 8942’s ability to inhibit *P. infestans* PP34 is attributed to hyphal coiling production around the pathogen’s hyphae and zoosporangia, a phenomenon often linked to physical disruption and nutrient competition during mycoparasitism. This behavior was observed in the interactions of *T. atroviride* against *P. infestans* [6] and *T. harzianum* against *Rhizoctonia solani* [42]. Differing from fungal phytopathogens, oomycetes lack chitin in their cell walls. Strain 8942’s strong inhibition against PP34 may involve non-chitinolytic enzymes. Our data revealed that the inhibition of 8942’s CFF and VOCs was >70% (Figure 1C), which highlights the functions of *Trichoderma*-derived secondary metabolites in suppressing *Phytophthora* growth and sporulation [6,7,43].

In addition to antagonism, 8942’s influence on tomato defense responses may be related to the reaction of plant tissues. In this case, physiological alternation and molecular effects against late blight should also be stressed, particularly in reactive oxygen species and key defense signaling pathways. Strain 8942 suppressed H_2_O_2_ accumulation and leaf necrosis through mitigation of oxidative damage accompanied by enhanced activities of oxalate oxidase and peroxidase (Figure 2C,D), which have not been systematically reported in *Trichoderma*–late blight interactions. In previous reports on biocontrol of bacterial and fungal phytopathogens, plant reactive oxygen species have played dual roles during pathogen infection, e.g., inducing tissue damage and defense-triggering signals [18,44]. The gene expression analysis showed that the treatment with 8942 rapidly upregulated *LOXA* (a gene associated with the JA pathway) and suppressed its negative regulator, *JAZ1*, in the early colonization process (Figure 2E). These changes are associated with interactions between 8942 treatment and JA-mediated ISR. This result supports previous studies on some non-pathogenic microbes activating JA-dependent pathways to reprogram phytohormone defense networks and thus improve stress tolerance [3,10,15].

In agriculture, the advantages of *Trichoderma* strains are also reflected in their capacities to promote plant growth [41]. Contreras-Cornejo et al. stated that the improvement of soil enzyme activities may further enhance the nutrition acquisition and stress tolerance of plants [27]. Our work found that strain 8942 increased the activities of urease and protease in rhizosphere soil, which might contribute to an improvement in nutrient absorption or plant growth. Auxin accumulation in roots also suggests that 8942 promoted root development (Figure 3A,B), which is possibly due to phytohormone regulation, as previously suggested [28,29]. The correlation between soil enzyme activities and plant IAA signaling (Figure 4A,C) suggests that 8942 might stimulate root architecture remodeling through synergistic interaction.

**Figure 6:**
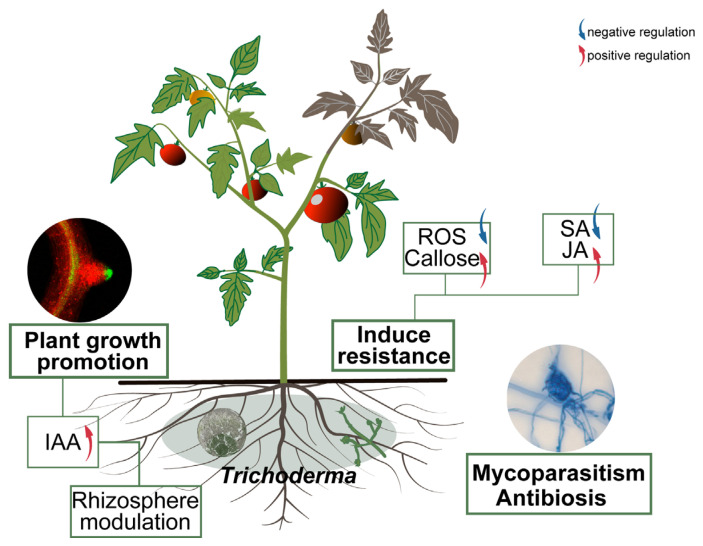
Mechanism of *T. paratroviride* 8942 in control of tomato late blight and promotion of plant growth. Arrows: blue indicating negative regulation; red indicating positive regulation.

Transcriptome analysis provided molecular insights into the gene expression reprogramming in strain 8942 during early interaction with tomato roots. Upon contacting the plant, 8942 differentially expressed 862 genes involved in transport, secondary metabolism, cell wall-degrading enzymes, and antioxidant defense (Figure 5A). Notably, the expression of many genes for glycoside hydrolases (the GH family) and chitinase was downregulated (Figure 5B,D), which is supposed to help 8942 evade plant immune recognition during initial colonization. It has been noted that restraint of CWDEs and toxin metabolism may prevent excessive tissue damage and achieve maximum avirulent colonization [10,45]. Meanwhile, a suite of oxidation–reduction genes in connection with peroxidases, catalases, glutathione S-transferases, and related enzymes were also downregulated (Figure 5B). This finding suggests that 8942 may actively mitigate provocation of a significant reactive oxygen species burst by the plant during early colonization, which accords with previous observations of *Trichoderma*–plant interactions [46,47]. Conversely, the induction of transporter (e.g., transmembrane transport and ion transport) genes indicates resource reallocation toward acquisition of plant nutrients. Such regulatory plasticity highlights *Trichoderma*’s evolutionary adaptation to switch between saprophytic and symbiotic states as an opportunistic symbiont, a process consistent with plant–symbiotic interactions [10,48]. The transcriptional shift observed in *Trichoderma*–tomato interaction reflects an evasive strategy to minimize host immune detection, which facilitates the establishment of a mutualistic relationship [7,10]. Our results provide strong evidence that *T. paratroviride* 8942 possesses abilities of direct pathogen suppression and antimicrobial metabolites, positive regulation of plant defenses, modulation of the rhizosphere environment, and promotion of plant growth (Figure 6).

In future studies, practical limitations, such as environmental variability, the influence of native soil microbiomes, and the need for field validation before agronomic application, should be considered.

## 5. Conclusions

In summary, *Trichoderma paratroviride* strain 8942 exhibits comprehensive antagonistic capabilities against the oomycete pathogen *Phytophthora infestans* causing late blight disease both in vitro and in pot experiments. We elucidated a multi-layered mechanism underlying the action of 8942 against *P. infestans* through dual-culture, metabolite profiling, plant defense response detection, and transcriptome analyses. Additionally, the strain also enhances plant growth through improvement of the rhizosphere environment and stimulation of plant root development. *Trichoderma paratroviride* 8942 is a potential biocontrol candidate that features in *P. infestans* suppression and plant growth promotion.

## Data Availability

The original contributions presented in this study are included in the article and Supplementary Materials. Further inquiries can be directed to the corresponding author. The RNA sequencing data reported in this paper have been deposited in the Genome Sequence Archive (Genomics, Proteomics & Bioinformatics 2025) in the National Genomics Data Center (Nucleic Acids Res 2025), China National Center for Bioinformation/Beijing Institute of Genomics, Chinese Academy of Sciences (GSA: CRA036052), and are publicly accessible at https://ngdc.cncb.ac.cn/gsa (accessed on 25 January 2026).

## Supplementary Materials

The following supporting information can be downloaded at https://www.mdpi.com/article/10.3390/jof12020096/s1: Figure S1: Disease severity grading for tomato late blight; Figure S2: Transcriptomic sampling design for *T. paratroviride* 8942–tomato interaction; Table S1: Physiological indices of tomato under different treatments; Table S2: Primers used for real-time qPCR. References [49,50,51,52,53,54,55,56,57] are cited in the Supplementary Materials.
