# Supplementary material for: Trichoderma paratroviride Strain 8942: Mechanisms of Phytophthora infestans Inhibition and Tomato Growth Promotion
Source: J Fungi (Basel). 2026 Jan 30;12(2):96. doi: 10.3390/jof12020096 (PMC12942607; doi:10.3390/jof12020096)
Supplement: Supplementary file 1 [file jof-12-00096-s001.zip › jof-4088222-supplementary-figures.pdf]

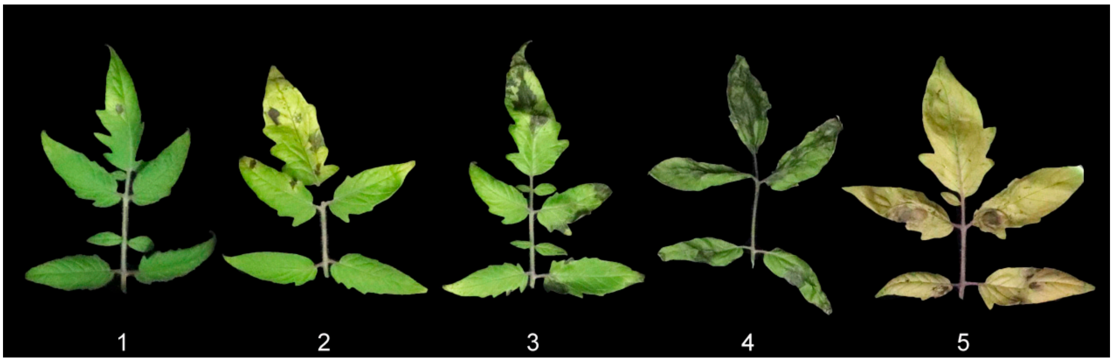

Figure S1. Disease severity grading for tomato late blight

Grade 1: small water-soaked lesions, lesion area < 5% of total leaf area. Grade 2: brown lesions, lesion area 5%–25%. Grade 3: brown lesions, lesion area 25%–50%. Grade 4: extensive brown lesions or wilted/curling leaves, lesion area 50%–75%. Grade 5: lesion area >75% or whole leaf withered.

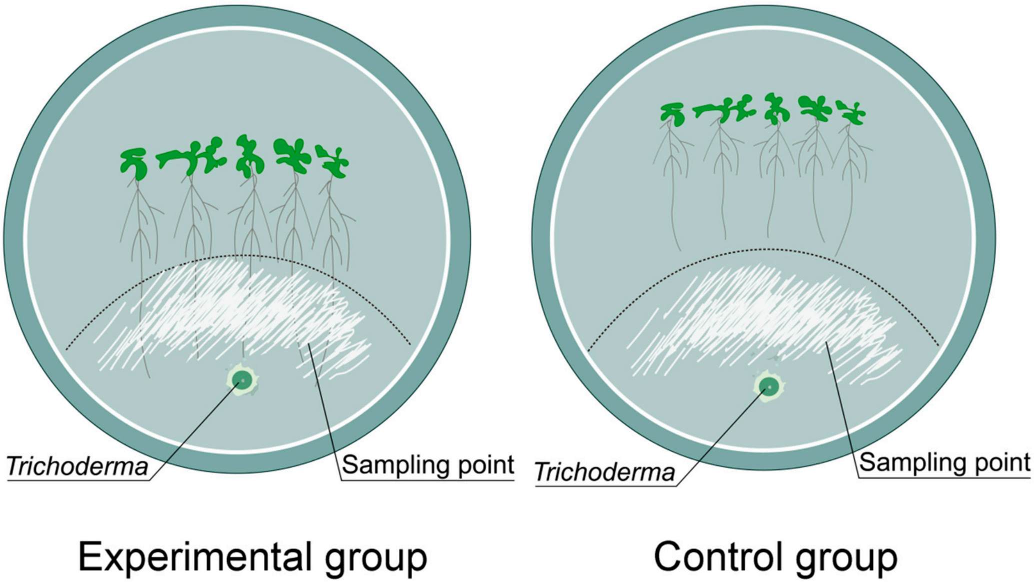

Figure S2. Transcriptomic sampling design for *T. paratroviride* 8942–tomato interaction

Experimental group: Tomato seeds germinated on Murashige & Skoog (MS) plates were co-cultured with *T. paratroviride* 8942. Sampling occurred 24 h after hyphal contact with tomato roots (Control: no contact). Samples were flash-frozen in liquid nitrogen and stored at −80°C. All treatments had 3 biological replicates.
