# Supplementary material for: Trichoderma paratroviride Strain 8942: Mechanisms of Phytophthora infestans Inhibition and Tomato Growth Promotion
Source: J Fungi (Basel). 2026 Jan 30;12(2):96. doi: 10.3390/jof12020096 (PMC12942607; doi:10.3390/jof12020096)
Supplement: Supplementary file 1 [file jof-12-00096-s001.zip › jof-4088222-supplementary-tables.pdf]

Table S1 Physiological indices of tomato under different treatments

Index	Biological function	References
Stomatal aperture	Protecting leaves from hyphal invasion	[49]
Callose	A physical barrier against hyphal invasion	[50]
H_2O_2	Inducing plant defense response	[51]
Oxalate oxidase	Decomposing oxalic acid	[52]
Peroxidase	Anti-oxidative damage	[53]

Table S2 Primers used for real-time qPCR

Gene	Pathway	Primer	Sequence（5’−3’）	References
LOXA	JA	F	TGCAGGTTACCTCCCAAATC	[54]
		R	AGCAGCGAGTGGTTCTTTGT	
JAZ1	JA	F	AAGACTCTGGGTTTCGCTGG	[55]
		R	CATCGACTCCATCTGTGGCA	
PR5	SA	F	GCAACAACTGTCCATACACC	[56]
		R	AGACTCCACCACAATCACC	
Actin2	Reference gene	F	TGGTCGGAATGGGACAGAAG	[57]
		R	CTCAGTCAGGAGAACAGGGT	
